# Baby survival in Zambia: stillbirth and neonatal death in a local hospital setting

**DOI:** 10.1186/s12884-019-2231-9

**Published:** 2019-03-12

**Authors:** Yasuhiro Miyoshi, Keiichi Matsubara, Norimi Takata, Yasunori Oka

**Affiliations:** 1 Zimba Mission Hospital, Zimba, Zambia; 2 Ehime University, Ehime, Japan; 3 Shikoku University, Tokushima, Japan

**Keywords:** Neonatal death, Stillbirth, Zambia, International classification of diseases - perinatal mortality (ICD-PM)

## Abstract

**Background:**

Globally, 2.6 million stillbirths occur every year. Of these, 98% occur in developing countries. According to the United Nations Children’s Fund, the neonatal mortality rate in Zambia in 2014 was 2.4%. In 2016, the World Health Organization released the International Classification of Diseases - Perinatal Mortality (ICD-PM) as a globally applicable and comparable system for the classification of the causes of perinatal deaths. However, data for developing countries are scarce. The aim of this study was to evaluate the rates and causes of stillbirths and neonatal deaths at a local hospital in Zimba, Zambia to identify opportunities for preventive interventions.

**Methods:**

All cases of stillbirths and neonatal deaths at Zimba Mission Hospital in Zambia in 2017 were included in this study. Outborn neonates who were transferred to the hospital and later died were also included in the study. Causes of stillbirths and neonatal deaths were analyzed and classified according to ICD-PM.

**Results:**

In total, 1754 babies were born via 1704 deliveries at the hospital, and 28 neonates were transferred to the hospital after birth. The total number of perinatal deaths was 75 (4.2%), with 7 deaths in the antepartum, 25 deaths in the intrapartum, and 43 deaths in the neonatal period. Most antepartum deaths (*n* = 5; 71.4%) were classified as fetal deaths of unspecified causes. Intrapartum deaths were due to acute intrapartum events (*n* = 21; 84.0%) or malformations, deformations, or chromosomal abnormalities (*n* = 4; 16.0%). Neonatal deaths were related primarily to complications from intrapartum events (*n* = 19; 44.2%); low birth weight or prematurity (*n* = 16; 37.2%); or infection (*n* = 3; 7.0%).

**Conclusions:**

Perinatal deaths were associated with acute intrapartum events and considered preventable in 40 cases (53.3%). Effective interventions to prevent perinatal deaths are needed.

## Background

According to World Health Organization (WHO), stillbirth is defined as death at delivery of a neonate weighing more than 1000 g or with a gestational age of 28 weeks or more [1]. The global stillbirth rate is estimated to be 18.4 per 1000 births, or 2.6 million stillbirths every year [2]. Stillbirth rates are lowest in Singapore and Finland (2 per 1000 births). It has been reported that 98% of stillbirths occur in developing countries [3]. The stillbirth rate in sub-Saharan Africa is 32.2 per 1000 births [4]. Because nearly half of deliveries in developing countries occur at home, under-reporting of stillbirths is a significant problem [5].

Neonatal death has been defined by WHO as “deaths among live births during the first 28 completed days of life” [6]. Neonatal mortality in Zambia was reported to be 24 per 1000 live births in 2014 according to the United Nations Children’s Fund (UNICEF) [7]. This high rate contrasts with a neonatal mortality of just 3 per 1000 live births in developed countries [3].

In sub-Saharan Africa, medical resources are limited and access to medical care is poorer, due to lack of infrastructure and a shortage of health care facilities. In rural Zambia, 42% of pregnant women deliver at home, whereas 56.3% deliver at health care facilities [8]. Pregnant women and their families are responsible for transportation to a health center. Doctors are usually not affiliated with health centers, and ultrasound assessment is not available. Registered nurses or midwives provide perinatal care. In the case of potential complications for the mother, fetus, or neonate, the nurse or midwife makes the patient transferred to a district hospital by ambulance or transport arranged by the patient’s family if no ambulance is available. Some hospitals and health centers are equipped with mothers’ shelters, where pregnant women living far from the facilities can stay free of charge while awaiting labor onset.

The high stillbirth and neonatal death rates in Africa can likely be reduced through more efficient use of limited resources. Therefore, preventable causes of deaths must be identified.

Global comparison is difficult because of the multiple classification systems used for perinatal deaths [9, 10]. WHO released the International Classification of Diseases - Perinatal Mortality (ICD-PM) as a globally applicable and comparable system in 2016 [11]; however, data for developing countries are scarce. The aim of this study was to evaluate the current status of stillbirths and neonatal deaths in Zambia using ICD-PM to classify and identify preventive interventions.

## Methods

### Study design and setting

This retrospective cross-sectional study was conducted at Zimba Mission Hospital in Southern Province, Zambia. This district hospital accepts patients referred from approximately 10 health centers in the catchment area, which serves a population of 98,000. The hospital is equipped with a mothers’ shelter to accommodate 50 pregnant women. High-risk patients, including those with breech presentation, multiple pregnancy, and hypertensive disorders of pregnancy, are supposed to be admitted to the hospital’s antenatal ward. There is 1 obstetrician but no pediatrician and no neonatologist affiliated with the hospital. Therefore, the obstetrician serves both mothers and neonates.

### Study population

All cases of stillbirths and neonatal deaths at Zimba Mission Hospital in Zambia from January through December 2017 were included in this study. Outborn neonates brought to the hospital were also included in the study.

### Data collection and definitions

Data were extracted from admission and delivery registers. Causes of stillbirths and neonatal deaths were analyzed with respect to clinical information and classified according to ICD-PM. Multi-disciplinary meetings with doctors, nurses, and midwives from the hospital and district health office were conducted to discuss perinatal deaths. There was no pathologist available, and no autopsies were performed. Gestational age was determined primarily using last menstrual period or fundal height, as ultrasound evaluation is not commonly performed during early pregnancy.

Simple statistical tests using absolute numbers were used to calculate percentages.

## Results

In 2017, there were 1754 neonate born through 1704 deliveries at Zimba Mission Hospital. There were 44 cases of twin pregnancy and 3 cases of triplet pregnancy. Another 28 neonates were transferred to the hospital after birth. Of the 1704 deliveries, 1400 (82.2%) were vaginal deliveries and 304 (17.8%) were cesarean deliveries. HIV infection was present in 81 mothers (4.8%).

Data were reviewed for a total of 75 perinatal deaths: 7 antepartum deaths, 25 intrapartum deaths, and 43 neonatal deaths (12 outborn). Of the 25 intrapartum deaths, fetal heartbeats were confirmed during the labor in 22 cases at the hospital or health centers. Fetal heartbeats were not confirmed during the labor in the other 3 cases. However those were considered intrapartum deaths due to the clinical history and fresh stillbirth in appearance. Of the 36 perinatal deaths in patients referred from outside facilities, 33 cases were referred during labor. Maternal complications (M1 to M4, ICD-PM) occurred in 28 of 33 cases, and time from referral to arrival was 30 min to 6 h.

Of the 7 antepartum deaths, 4 (57.1%) occurred after 37 weeks’ gestation, 2 (28.6%) before 37 weeks’ gestation, and 1 (14.3%) at unknown gestational age. There was 1 case of antepartum death in a twin pregnancy (one of the twin). Of the 25 intrapartum deaths, 17 (68.0%) occurred after 37 weeks’ gestation, 7 (28.0%) before 37 weeks’ gestation, and 1 (4.0%) at unknown gestational age. There were 2 cases of intrapartum deaths in twin pregnancies (one of the twin). Of the 43 neonates who died, 17 (39.6%) had been born after 37 weeks’ gestation, 15 (34.9%) before 37 weeks’ gestation, and 11 (25.6%) at unknown gestational age. Neonatal death occurred in 2 twins (one of the twin) and 1 triplet (two of the triplet). Table 1 shows perinatal causes of death by maternal condition for all perinatal deaths.

**Table 1:** Causes of perinatal deaths according to ICD-PM

Maternal Condition	M1: Complications of placenta, cord and membranes	M2: Maternal complications of pregnancy	M3: Other complications of labor and pregnancy	M4: Maternal medical and surgical conditions	M5: No maternal condition identified	Total (%)
Antepartum death (A)						
A1: Congenital malformations, deformations and chromosomal abonormalities	0	0	0	0	1	1 (14.3)
A2: Infection	0	0	0	0	0	0 (0.0)
A3: Antepartum hypoxia	0	0	0	1	0	1 (14.3)
A4: Other specified antepartum disorder	0	0	0	0	0	0 (0.0)
A5: Disorders related to fetal growth	0	0	0	0	0	0 (0.0)
A6: Fetal death of unspecified causes	0	1	1	1	2	5 (71.4)
Total (%)	0	1 (14.3)	1(14.3)	2 (28.6)	3 (42.9)	7
Intrapartum death (I)						
I1: Congenital malformations, deformations and chromosomal abnormalities	0	0	0	0	4	4 (16.0)
I2: Birth trauma	0	0	0	0	0	0 (0.0)
I3: Acute intrapartum event	8	2	9	1	1	21 (84.0)
I4: Infection	0	0	0	0	0	0 (0.0)
I5: Other specified intrapartum disorder	0	0	0	0	0	0 (0.0)
I6: Disorders related to fetal growth	0	0	0	0	0	0 (0.0)
I7: Intrapartum death of unspecified cause	0	0	0	0	0	0 (0.0)
Total (%)	8 (32.0)	2 (8.0)	9 (36.0)	1 (4.0)	5 (20.0)	25
Neonatal death (N)						
N1: Congenital malformations, deformations and chromosomal abnormalities	0	0	0	0	1	1 (2.3)
N2: Disorders related to fetal growth	0	0	0	0	0	0 (0.0)
N3: Birth trauma	0	0	0	0	0	0 (0.0)
N4: Complications of intrapartum events	1	3	7	0	8	19 (44.2)
N5: Convulsions and disorders of cerebral status	0	0	0	0	0	0 (0.0)
N6: Infection	0	0	1	0	2	3 (7.0)
N7: Respiratory and cardiovascular disorders	0	0	0	0	2	2 (4.7)
N8: Other neonatal conditions	0	0	0	0	0	0 (0.0)
N9: Low birth weight and prematurity	0	1	15	0	0	16 (37.2)
N10: Miscellaneous	0	0	0	0	0	0 (0.0)
N11: Neonatal deaths of unspecified cause	0	0	0	0	2	2 (4.7)
Total (%)	1 (2.3)	4 (9.3)	23 (53.5)	0	15 (34.9)	43

Antepartum deaths were classified as fetal deaths of unspecified cause (*n* = 5, 71.4%); congenital malformations, deformations and chromosomal abnormalities (*n* = 1, 14.3%); and antepartum hypoxia (preeclampsia [*n* = 1, 14.3%]). The 3 (60%) fetal deaths due to unspecified causes were associated with multiple pregnancy, malpresentation before labor, and maternal HIV infection.

Intrapartum deaths often followed acute intrapartum events (*n* = 21, 84.0%), and 13 cases (61.9%) were referred from outside health care facilities. Most intrapartum deaths (*n* = 20, 95.2%) occurred in mothers with pre-existing conditions. Of the 9 women (42.9%) who experienced other complications of labor and delivery prior to intrapartum death, 7 (77.8%) experienced obstructed labor due to malposition, malpresentation, or disproportion, and 2 (22.2%) experienced uterine rupture. Of the 2 cases of intrapartum death associated with breech presentation, 1 was transferred from the mothers’ shelter after fetal death. That woman had a rapidly progressing labor and the fetal head became trapped in the birth canal. The second most frequent maternal condition associated with acute intrapartum events was complication of the placenta, cord, or membrane (*n* = 8, 38.1%). Of these, 6 cases (75.0%) had prolapsed cords, and 2 cases (25.0%) had nuchal cords. All 6 cases of cord prolapse were referred from outside facilities. Other associated maternal conditions complicated 2 pregnancies (9.5%): 1 spontaneous preterm labor and 1 twin pregnancy. Preeclampsia occurred in 1 patient (4.8%). Of the intrapartum deaths due to malformations, deformations, or chromosomal abnormalities (*n* = 4, 16.0%). Those 4 consist of a conjoined twin (it was considered a single stillbirth), short extremities with severe polyhydramnios (suspected thanatophoric dysplasia), anencephaly, and holoprosencephaly.

Among the 43 neonatal deaths (2.5% of live births), 39 deaths (90.7%) occurred within 1 week of birth. Neonatal deaths generally followed complications of intrapartum events (*n* = 19, 44.2%) of low birth weight or prematurity (*n* = 16, 37.2%) or infection (*n* = 3, 7.0%). More than half (*n* = 11, 57.9%) of neonatal deaths associated with intrapartum complications occurred among mothers with known conditions. Ten (52.6%) cases of neonatal death due to intrapartum complications had been referred from outside health care facilities: 8 maternal and 2 neonatal referrals. One neonatal death occurred during breech delivery due to entrapment of the head. Of the 16 neonates with low birth weight or prematurity who died, 6 (37.5%) weighed less than 1 kg at birth, and 9 (56.3%) had been born at home or outside health centers. All neonatal deaths due to infection occurred within 2 days of birth.

There were no maternal deaths associated with perinatal deaths.

## Discussion

The rate of stillbirths occurring during the intrapartum period during our study was strikingly high, at 78.1%. This rate contrasts with an estimated global rate of 26% [12]. Pilot testing of the ICD-PM database has shown the rate of intrapartum stillbirths to be 9.4% in the United Kingdom (high-income country) and 17.8% in South Africa (middle-income country) [13].

Perinatal death of a baby born after 28 weeks gestation due to an acute intrapartum event is usually defined as preventable [12]. Thus, 21 stillbirths (65.6% of total stillbirths) and 19 neonatal deaths (44.2% of total neonatal deaths) in our study would be considered preventable. Most of these cases were referred from outside health care facilities or reached the hospital late from home. Had maternal risks been assessed prior to labor and had mothers presented to the hospital earlier, those perinatal deaths may have been prevented.

Education of nurses and midwives regarding emergent obstetric care at health centers and hospitals is the primary way in which to address preventable stillbirths and neonatal deaths. Nurses and midwives need to assess patient risk and conduct timely referral. Furthermore, nurses and midwives at hospitals need to accurately assess patient condition, contact the obstetrician in a timely manner, and manage problems before obstetrician arrival.

There were 3 perinatal deaths due to entrapment of the head that resulted from lack of training. Another 6 cases of stillbirth were referred for cord prolapse. Staff at health centers should have been trained to push up the presenting part to prevent umbilical cord compression and refer these patients earlier to prevent those deaths. Therefore, training on breech delivery and management of cord prolapse is essential. Education on neonatal resuscitation is provided daily to staff working in the maternity ward.

Continuity of staff education is crucial to minimizing perinatal deaths. Although cardiotocogram (CTG) monitors are rarely available in sub-Saharan African, we are fortunate to have several CTG monitors at our hospital. CTG monitors are supposed to be used on admission for every patient and continued as needed. However, abnormal signs on CTG, such as late deceleration, are often missed and monitoring is not continuous, even for patients at risk for preeclampsia. Seven babies (9.3%) in this study were impacted by this failure to monitor appropriately. Earlier detection by staff may have led to cesarean section and prevented perinatal death.

In addition to improving perinatal care at health care facilities, appropriate education for pregnant women is crucially important to reduce the rate of stillbirths and neonatal deaths. Antenatal visits and hospital delivery need to be emphasized to help women with high-risk pregnancies. It is well-known that longer travel time from health centers to the district hospital is associated with adverse neonatal outcomes [14]. Thus, it is important to reduce the transport delay by improving referral systems. In 2017, emergent obstetrical cases at a rural health center required an ambulance from the hospital be sent to transport patients. Thus, there were long delays for patients to reach the hospital. Since that time, some rural health centers have obtained their own ambulances so that the patients can reach the hospital earlier. The mothers’ shelter near the hospital also plays an important role.

The number of deliveries at our hospital has tripled since 2011, and accurate data regarding perinatal deaths has only been available since 2017. Thus, the annual rates of perinatal deaths cannot be compared over time. Perinatal deaths are related to delays in medical care. Thus, increasing the proportion of deliveries at the hospital may contribute to an overall reduction in perinatal deaths. Weekly clinical rounds at the mothers’ shelter were introduced after in December 2017 after some cases of intrauterine fetal death occurred there. High-risk cases such as preeclampsia, multiple pregnancy, and abnormal presentation or lie are now identified and these have not been associated with perinatal death since that time.

The second leading cause of neonatal death in this study was low birth weight or prematurity (*n* = 16, 37.2%), which are related to poor medical resources, lack of neonatal care specialists, and delay in accepting appropriate medical care. Had those neonates been born in the hospital, outcomes may have differed. Although our hospital has nasal bubble continuous positive airway pressure to support respiration, we do not have ventilators or surfactant like hospitals in developed countries.

The third leading cause of neonatal deaths was infection (*n* = 3, 7.0%). Bacterial infection with group B streptococcus (GBS) from the birth canal may have contributed to these neonatal deaths. The incidence of newborn GBS sepsis in developing countries is estimated to be up to 3 per 1000 live births [15]. Although GBS and *Chlamydia trachomatis* antenatal screening are performed for all pregnant women in developed countries, no such screening is performed in Zambia. Thus, GBS cannot be treated at labor onset, as if done in developed countries. Finding solutions to reduce neonatal deaths caused by infection is an objective for future work.

Comparison of our data with those from other studies is difficult because the study populations differ. One study in a tertiary care center in Sri Lanka showed that antepartum hypoxia was the most common cause of antepartum death (41.9%), whereas congenital malformation was the most common cause of intrapartum death (71.4%) and neonatal death (44.2%) [16].

There are some limitations for the current study. Identification of causes of stillbirths and neonatal deaths is complex. In Zambia, thorough investigation cannot be performed due to lack of resources and absence of a neonatal specialist. For example, we were unable to evaluate the impact of infectious diseases such as syphilis, toxoplasma, cytomegalovirus, and rubella due to the poor laboratory resources. Estimates suggest that infection contributes to nearly half of stillbirths in developing countries [5]. In areas where syphilis is highly prevalent, up to half of all stillbirths may be caused by this infection alone [17]. Maternal anemia is also known to be associated with perinatal death [18]; however, routine hemoglobin testing is not available for all pregnant women in developing countries. Thus, the impact of maternal anemia on perinatal mortality was not evaluated in this study.

## Conclusion

More than half of perinatal deaths resulted from acute intrapartum events that could have been prevented. Effective interventions, including better assessment of maternal risk and timely hospital referral could contribute to the reduction of perinatal deaths in Zambia.

## Abbreviations

CTG: Cardiotocogram
GBS: Group B streptococcus
ICD-PM: International classification of diseases - perinatal mortality
UNICEF: United Nations’ Children’s Fund
WHO: World Health Organization
